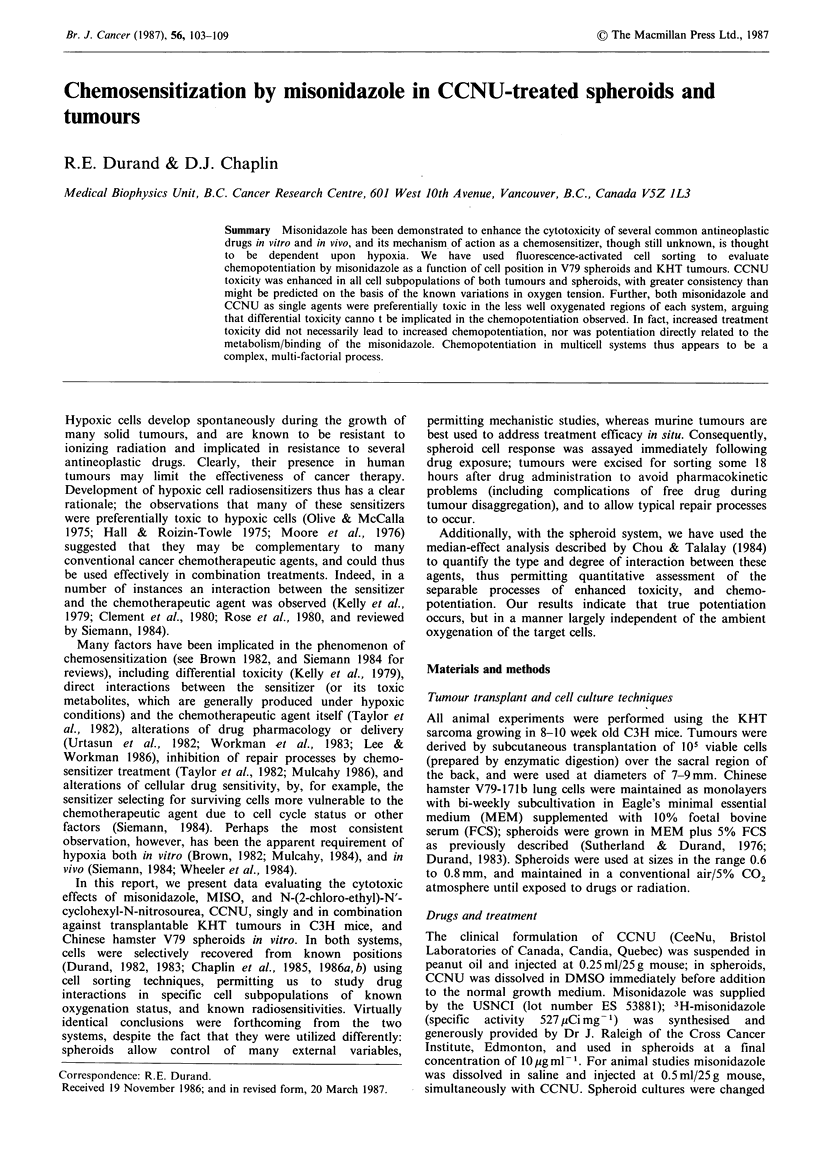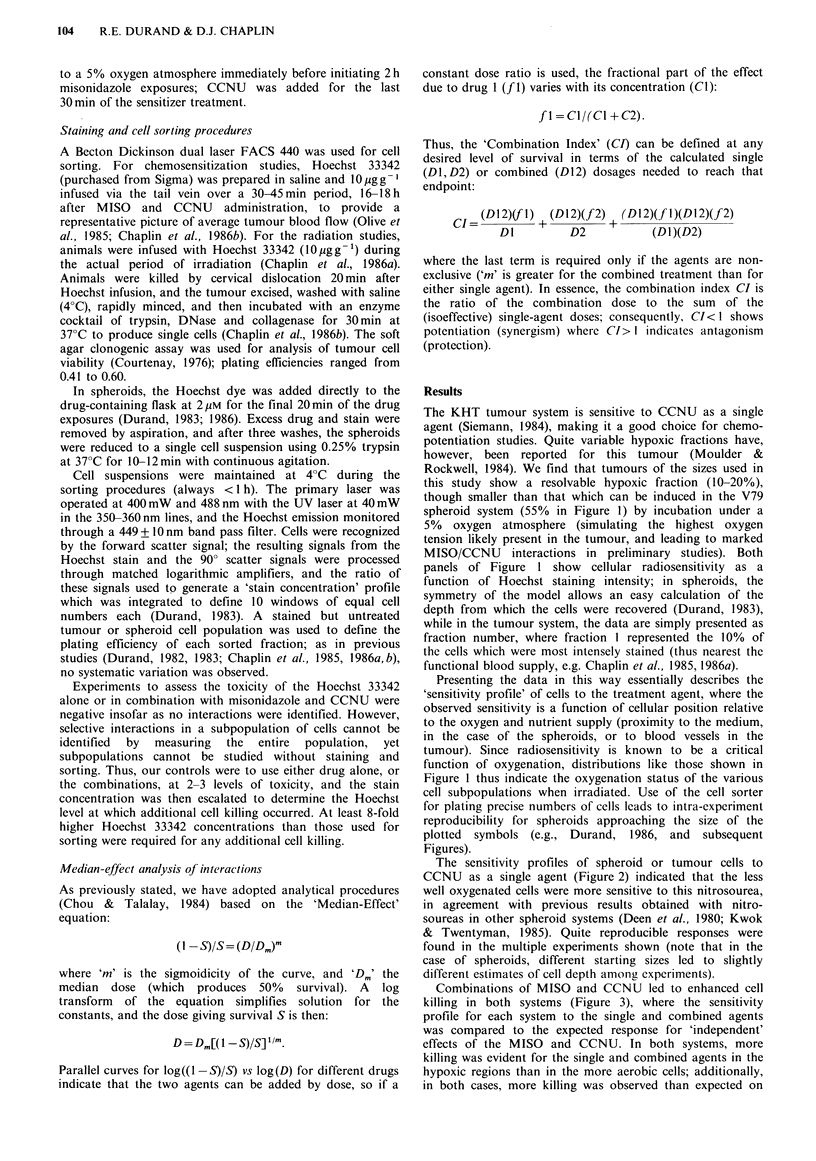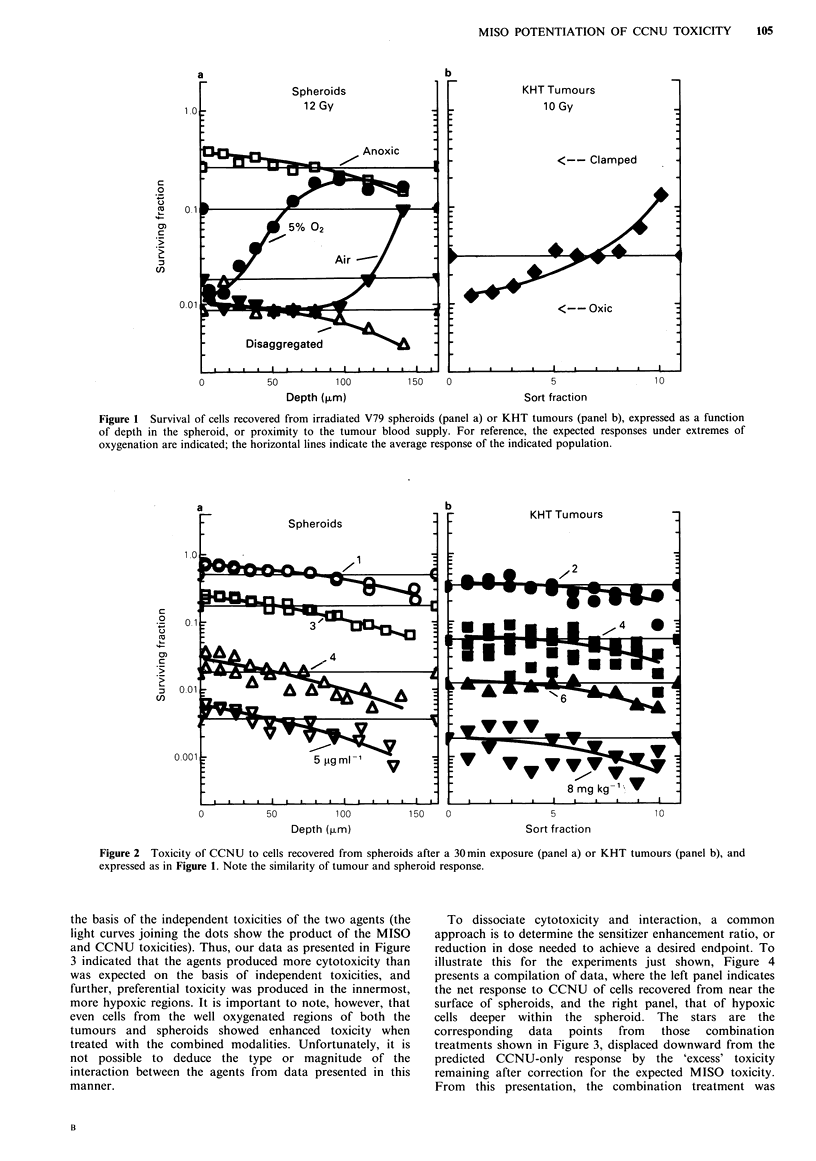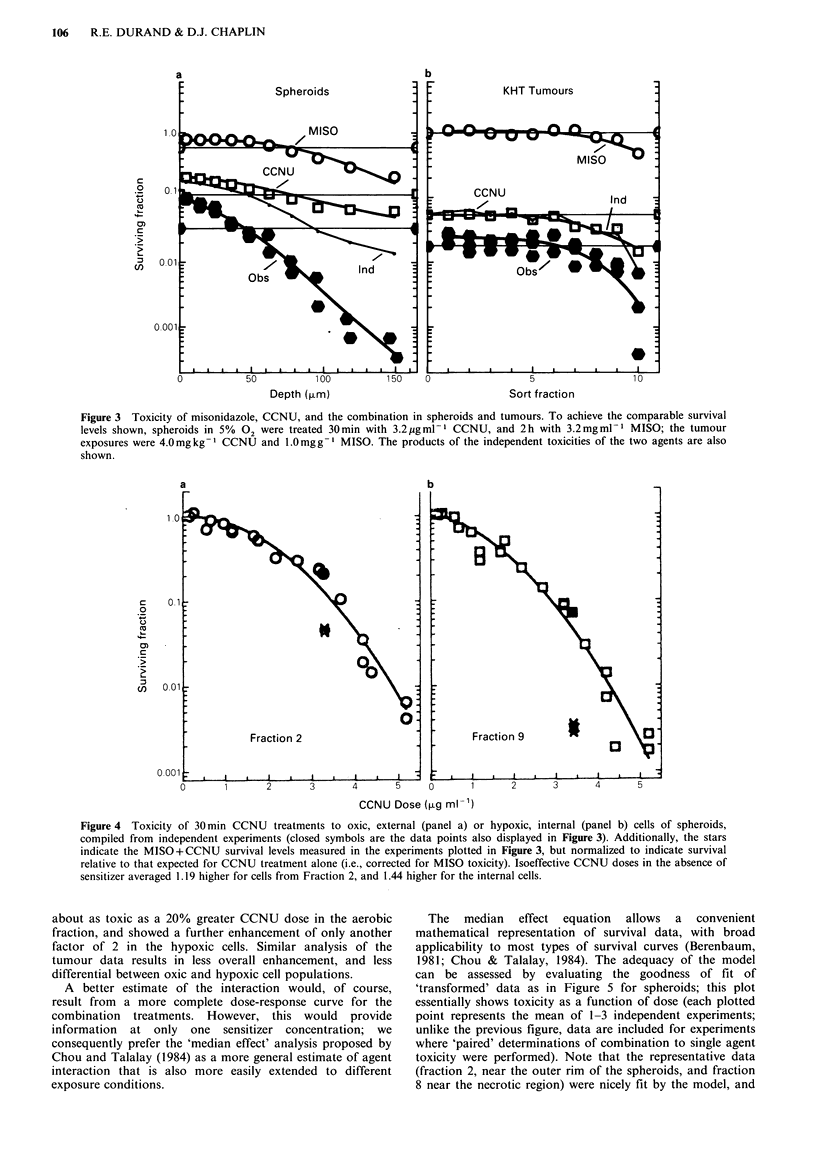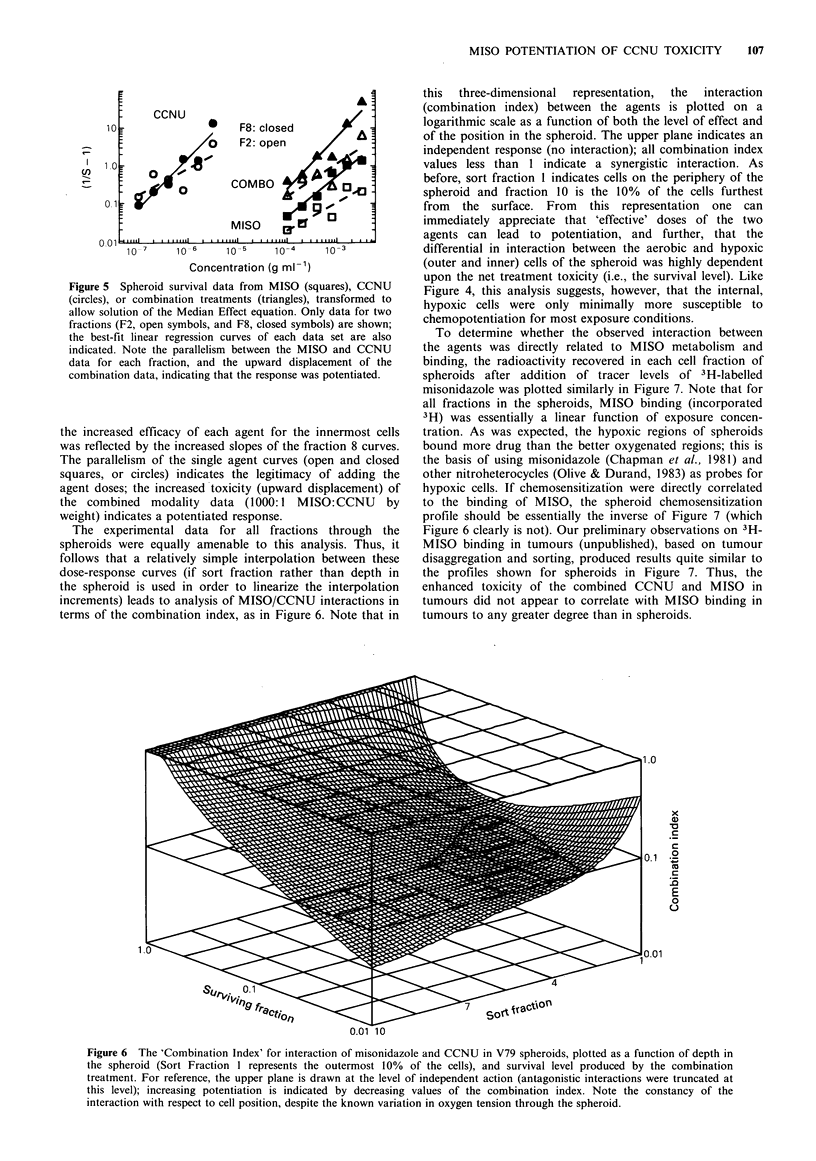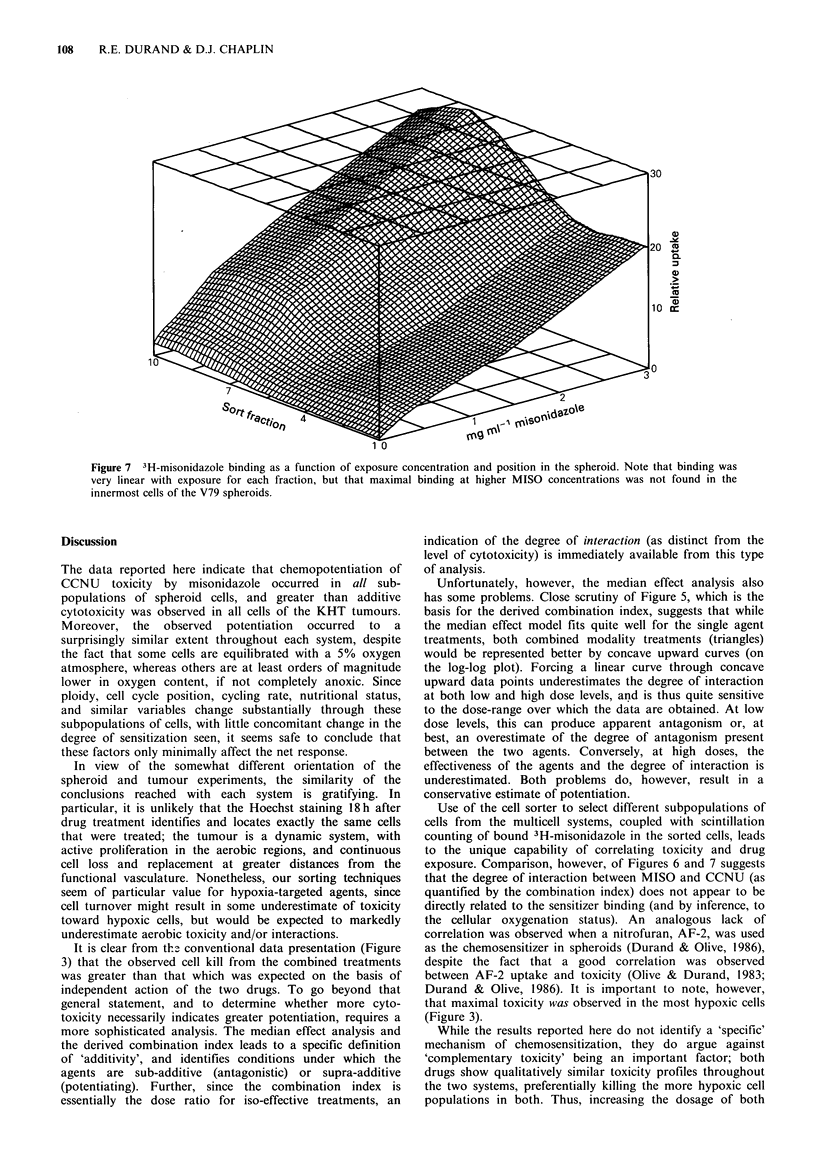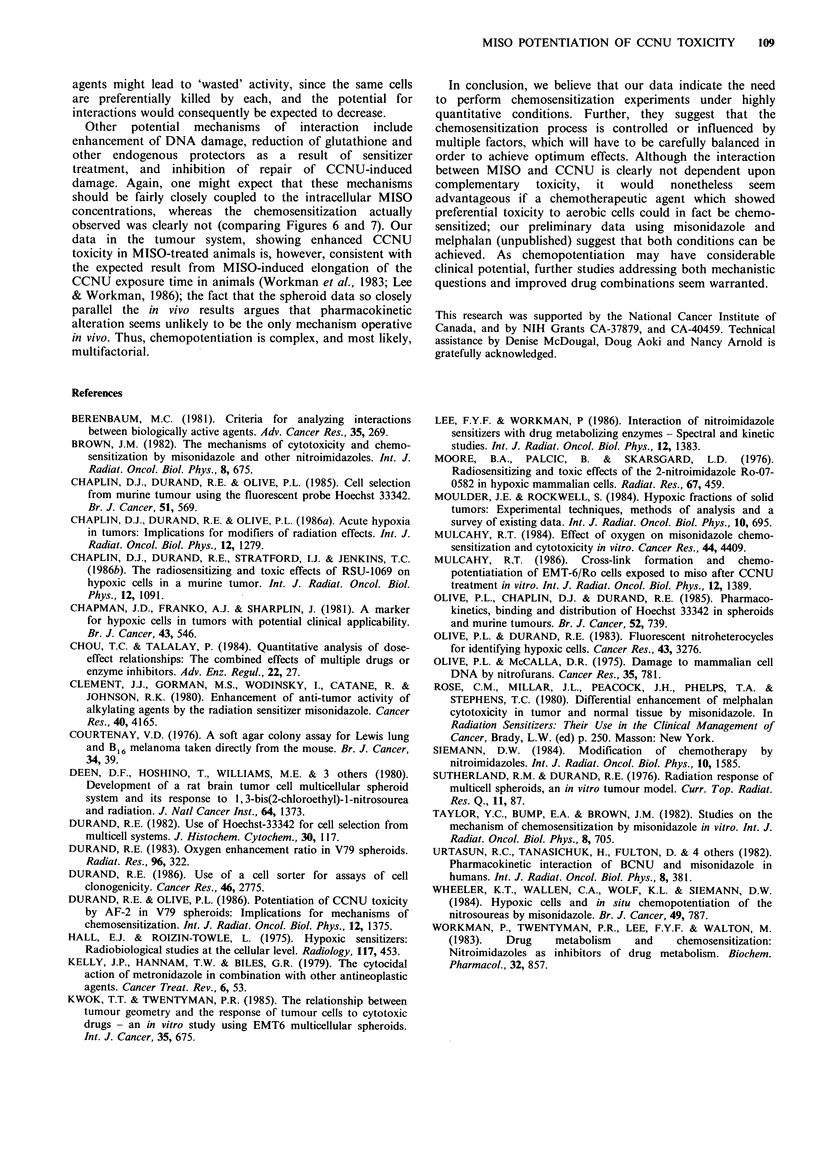# Chemosensitization by misonidazole in CCNU-treated spheroids and tumours.

**DOI:** 10.1038/bjc.1987.165

**Published:** 1987-08

**Authors:** R. E. Durand, D. J. Chaplin

**Affiliations:** Medical Biophysics Unit, B.C. Cancer Research Centre, Vancouver, Canada.

## Abstract

Misonidazole has been demonstrated to enhance the cytotoxicity of several common antineoplastic drugs in vitro and in vivo, and its mechanism of action as a chemosensitizer, though still unknown, is thought to be dependent upon hypoxia. We have used fluorescence-activated cell sorting to evaluate chemopotentiation by misonidazole as a function of cell position in V79 spheroids and KHT tumours. CCNU toxicity was enhanced in all cell subpopulations of both tumours and spheroids, with greater consistency than might be predicted on the basis of the known variations in oxygen tension. Further, both misonidazole and CCNU as single agents were preferentially toxic in the less well oxygenated regions of each system, arguing that differential toxicity cannot be implicated in the chemopotentiation observed. In fact, increased treatment toxicity did not necessarily lead to increased chemopotentiation, nor was potentiation directly related to the metabolism/binding of the misonidazole. Chemopotentiation in multicell systems thus appears to be a complex, multi-factorial process.


Br. J. Cancer (1987), 56, 103–109 © The Macmillan Press Ltd., 1987

### Chemosensitization by misonidazole in CCNU-treated spheroids and tumours

R.E. Durand & D.J. Chaplin

*Medical Biophysics Unit, B.C. Cancer Research Centre, 601 West 10th Avenue, Vancouver, B.C., Canada V5Z 1L3*

**Summary** Misonidazole has been demonstrated to enhance the cytotoxicity of several common antineoplastic drugs *in vitro* and *in vivo*, and its mechanism of action as a chemosensitizer, though still unknown, is thought to be dependent upon hypoxia. We have used fluorescence-activated cell sorting to evaluate chemopotentiation by misonidazole as a function of cell position in V79 spheroids and KHT tumours. CCNU toxicity was enhanced in all cell subpopulations of both tumours and spheroids, with greater consistency than might be predicted on the basis of the known variations in oxygen tension. Further, both misonidazole and CCNU as single agents were preferentially toxic in the less well oxygenated regions of each system, arguing that differential toxicity canno t be implicated in the chemopotentiation observed. In fact, increased treatment toxicity did not necessarily lead to increased chemopotentiation, nor was potentiation directly related to the metabolism/binding of the misonidazole. Chemopotentiation in multicell systems thus appears to be a complex, multi-factorial process.

Hypoxic cells develop spontaneously during the growth of many solid tumours, and are known to be resistant to ionizing radiation and implicated in resistance to several antineoplastic drugs. Clearly, their presence in human tumours may limit the effectiveness of cancer therapy. Development of hypoxic cell radiosensitizers thus has a clear rationale; the observations that many of these sensitizers were preferentially toxic to hypoxic cells (Olive & McCalla 1975; Hall & Roizin-Towle 1975; Moore *et al.*, 1976) suggested that they may be complementary to many conventional cancer chemotherapeutic agents, and could thus be used effectively in combination treatments. Indeed, in a number of instances an interaction between the sensitizer and the chemotherapeutic agent was observed (Kelly *et al.*, 1979; Clement *et al.*, 1980; Rose *et al.*, 1980, and reviewed by Siemann, 1984).

Many factors have been implicated in the phenomenon of chemosensitization (see Brown 1982, and Siemann 1984 for reviews), including differential toxicity (Kelly *et al.*, 1979), direct interactions between the sensitizer (or its toxic metabolites, which are generally produced under hypoxic conditions) and the chemotherapeutic agent itself (Taylor *et al.*, 1982), alterations of drug pharmacology or delivery (Urtasun *et al.*, 1982; Workman *et al.*, 1983; Lee & Workman 1986), inhibition of repair processes by chemosensitizer treatment (Taylor *et al.*, 1982; Mulcahy 1986), and alterations of cellular drug sensitivity, by, for example, the sensitizer selecting for surviving cells more vulnerable to the chemotherapeutic agent due to cell cycle status or other factors (Siemann, 1984). Perhaps the most consistent observation, however, has been the apparent requirement of hypoxia both *in vitro* (Brown, 1982; Mulcahy, 1984), and *in vivo* (Siemann, 1984; Wheeler *et al.*, 1984).

In this report, we present data evaluating the cytotoxic effects of misonidazole, MISO, and N-(2-chloro-ethyl)-N′-cyclohexyl-N-nitrosourea, CCNU, singly and in combination against transplantable KHT tumours in C3H mice, and Chinese hamster V79 spheroids *in vitro*. In both systems, cells were selectively recovered from known positions (Durand, 1982, 1983; Chaplin *et al.*, 1985, 1986*a*,*b*) using cell sorting techniques, permitting us to study drug interactions in specific cell subpopulations of known oxygenation status, and known radiosensitivities. Virtually identical conclusions were forthcoming from the two systems, despite the fact that they were utilized differently: spheroids allow control of many external variables, permitting mechanistic studies, whereas murine tumours are best used to address treatment efficacy *in situ*. Consequently, spheroid cell response was assayed immediately following drug exposure; tumours were excised for sorting some 18 hours after drug administration to avoid pharmacokinetic problems (including complications of free drug during tumour disaggregation), and to allow typical repair processes to occur.

Additionally, with the spheroid system, we have used the median-effect analysis described by Chou & Talalay (1984) to quantify the type and degree of interaction between these agents, thus permitting quantitative assessment of the separable processes of enhanced toxicity, and chemopotentiation. Our results indicate that true potentiation occurs, but in a manner largely independent of the ambient oxygenation of the target cells.

### Materials and methods

### *Tumour transplant and cell culture techniques*

All animal experiments were performed using the KHT sarcoma growing in 8–10 week old C3H mice. Tumours were derived by subcutaneous transplantation of $10^5$ viable cells (prepared by enzymatic digestion) over the sacral region of the back, and were used at diameters of 7–9 mm. Chinese hamster V79-171b lung cells were maintained as monolayers with bi-weekly subcultivation in Eagle's minimal essential medium (MEM) supplemented with 10% foetal bovine serum (FCS); spheroids were grown in MEM plus 5% FCS as previously described (Sutherland & Durand, 1976; Durand, 1983). Spheroids were used at sizes in the range 0.6 to 0.8 mm, and maintained in a conventional air/5% $CO_2$ atmosphere until exposed to drugs or radiation.

### *Drugs and treatment*

The clinical formulation of CCNU (CeeNu, Bristol Laboratories of Canada, Candia, Quebec) was suspended in peanut oil and injected at 0.25 ml/25 g mouse; in spheroids, CCNU was dissolved in DMSO immediately before addition to the normal growth medium. Misonidazole was supplied by the USNCI (lot number ES 53881); $^3$H-misonidazole (specific activity 527 $\mu$Ci mg$^{-1}$) was synthesised and generously provided by Dr J. Raleigh of the Cross Cancer Institute, Edmonton, and used in spheroids at a final concentration of 10 $\mu$g ml$^{-1}$. For animal studies misonidazole was dissolved in saline and injected at 0.5 ml/25 g mouse, simultaneously with CCNU. Spheroid cultures were changed

Correspondence: R.E. Durand.
Received 19 November 1986; and in revised form, 20 March 1987.

to a 5% oxygen atmosphere immediately before initiating 2 h misonidazole exposures; CCNU was added for the last 30 min of the sensitizer treatment.

*Staining and cell sorting procedures*

A Becton Dickinson dual laser FACS 440 was used for cell sorting. For chemosensitization studies, Hoechst 33342 (purchased from Sigma) was prepared in saline and $10\,\mu g\,g^{-1}$ infused via the tail vein over a 30–45 min period, 16–18 h after MISO and CCNU administration, to provide a representative picture of average tumour blood flow (Olive *et al.*, 1985; Chaplin *et al.*, 1986*b*). For the radiation studies, animals were infused with Hoechst 33342 ($10\,\mu g\,g^{-1}$) during the actual period of irradiation (Chaplin *et al.*, 1986*a*). Animals were killed by cervical dislocation 20 min after Hoechst infusion, and the tumour excised, washed with saline (4°C), rapidly minced, and then incubated with an enzyme cocktail of trypsin, DNase and collagenase for 30 min at 37°C to produce single cells (Chaplin *et al.*, 1986*b*). The soft agar clonogenic assay was used for analysis of tumour cell viability (Courtenay, 1976); plating efficiencies ranged from 0.41 to 0.60.

In spheroids, the Hoechst dye was added directly to the drug-containing flask at $2\,\mu M$ for the final 20 min of the drug exposures (Durand, 1983; 1986). Excess drug and stain were removed by aspiration, and after three washes, the spheroids were reduced to a single cell suspension using 0.25% trypsin at 37°C for 10–12 min with continuous agitation.

Cell suspensions were maintained at 4°C during the sorting procedures (always <1 h). The primary laser was operated at 400 mW and 488 nm with the UV laser at 40 mW in the 350–360 nm lines, and the Hoechst emission monitored through a $449 \pm 10$ nm band pass filter. Cells were recognized by the forward scatter signal; the resulting signals from the Hoechst stain and the 90° scatter signals were processed through matched logarithmic amplifiers, and the ratio of these signals used to generate a 'stain concentration' profile which was integrated to define 10 windows of equal cell numbers each (Durand, 1983). A stained but untreated tumour or spheroid cell population was used to define the plating efficiency of each sorted fraction; as in previous studies (Durand, 1982, 1983; Chaplin *et al.*, 1985, 1986*a,b*), no systematic variation was observed.

Experiments to assess the toxicity of the Hoechst 33342 alone or in combination with misonidazole and CCNU were negative insofar as no interactions were identified. However, selective interactions in a subpopulation of cells cannot be identified by measuring the entire population, yet subpopulations cannot be studied without staining and sorting. Thus, our controls were to use either drug alone, or the combinations, at 2–3 levels of toxicity, and the stain concentration was then escalated to determine the Hoechst level at which additional cell killing occurred. At least 8-fold higher Hoechst 33342 concentrations than those used for sorting were required for any additional cell killing.

*Median-effect analysis of interactions*

As previously stated, we have adopted analytical procedures (Chou & Talalay, 1984) based on the 'Median-Effect' equation:

$$(1-S)/S=(D/D_m)^m$$

where '$m$' is the sigmoidicity of the curve, and '$D_m$' the median dose (which produces 50% survival). A log transform of the equation simplifies solution for the constants, and the dose giving survival $S$ is then:

$$D=D_m[(1-S)/S]^{1/m}.$$

Parallel curves for $\log((1-S)/S)$ *vs* $\log(D)$ for different drugs indicate that the two agents can be added by dose, so if a constant dose ratio is used, the fractional part of the effect due to drug 1 ($f1$) varies with its concentration ($C1$):

$$f1=C1/(C1+C2).$$

Thus, the 'Combination Index' ($CI$) can be defined at any desired level of survival in terms of the calculated single ($D1, D2$) or combined ($D12$) dosages needed to reach that endpoint:

$$CI=\frac{(D12)(f1)}{D1}+\frac{(D12)(f2)}{D2}+\frac{(D12)(f1)(D12)(f2)}{(D1)(D2)}$$

where the last term is required only if the agents are non-exclusive ('$m$' is greater for the combined treatment than for either single agent). In essence, the combination index $CI$ is the ratio of the combination dose to the sum of the (isoeffective) single-agent doses; consequently, $CI<1$ shows potentiation (synergism) where $CI>1$ indicates antagonism (protection).

### Results

The KHT tumour system is sensitive to CCNU as a single agent (Siemann, 1984), making it a good choice for chemopotentiation studies. Quite variable hypoxic fractions have, however, been reported for this tumour (Moulder & Rockwell, 1984). We find that tumours of the sizes used in this study show a resolvable hypoxic fraction (10–20%), though smaller than that which can be induced in the V79 spheroid system (55% in Figure 1) by incubation under a 5% oxygen atmosphere (simulating the highest oxygen tension likely present in the tumour, and leading to marked MISO/CCNU interactions in preliminary studies). Both panels of Figure 1 show cellular radiosensitivity as a function of Hoechst staining intensity; in spheroids, the symmetry of the model allows an easy calculation of the depth from which the cells were recovered (Durand, 1983), while in the tumour system, the data are simply presented as fraction number, where fraction 1 represented the 10% of the cells which were most intensely stained (thus nearest the functional blood supply, e.g. Chaplin *et al.*, 1985, 1986*a*).

Presenting the data in this way essentially describes the 'sensitivity profile' of cells to the treatment agent, where the observed sensitivity is a function of cellular position relative to the oxygen and nutrient supply (proximity to the medium, in the case of the spheroids, or to blood vessels in the tumour). Since radiosensitivity is known to be a critical function of oxygenation, distributions like those shown in Figure 1 thus indicate the oxygenation status of the various cell subpopulations when irradiated. Use of the cell sorter for plating precise numbers of cells leads to intra-experiment reproducibility for spheroids approaching the size of the plotted symbols (e.g., Durand, 1986, and subsequent Figures).

The sensitivity profiles of spheroid or tumour cells to CCNU as a single agent (Figure 2) indicated that the less well oxygenated cells were more sensitive to this nitrosourea, in agreement with previous results obtained with nitrosoureas in other spheroid systems (Deen *et al.*, 1980; Kwok & Twentyman, 1985). Quite reproducible responses were found in the multiple experiments shown (note that in the case of spheroids, different starting sizes led to slightly different estimates of cell depth among experiments).

Combinations of MISO and CCNU led to enhanced cell killing in both systems (Figure 3), where the sensitivity profile for each system to the single and combined agents was compared to the expected response for 'independent' effects of the MISO and CCNU. In both systems, more killing was evident for the single and combined agents in the hypoxic regions than in the more aerobic cells; additionally, in both cases, more killing was observed than expected on

**Figure 1** Survival of cells recovered from irradiated V79 spheroids (panel a) or KHT tumours (panel b), expressed as a function of depth in the spheroid, or proximity to the tumour blood supply. For reference, the expected responses under extremes of oxygenation are indicated; the horizontal lines indicate the average response of the indicated population.

**Figure 2** Toxicity of CCNU to cells recovered from spheroids after a 30 min exposure (panel a) or KHT tumours (panel b), and expressed as in **Figure 1**. Note the similarity of tumour and spheroid response.

the basis of the independent toxicities of the two agents (the light curves joining the dots show the product of the MISO and CCNU toxicities). Thus, our data as presented in Figure 3 indicated that the agents produced more cytotoxicity than was expected on the basis of independent toxicities, and further, preferential toxicity was produced in the innermost, more hypoxic regions. It is important to note, however, that even cells from the well oxygenated regions of both the tumours and spheroids showed enhanced toxicity when treated with the combined modalities. Unfortunately, it is not possible to deduce the type or magnitude of the interaction between the agents from data presented in this manner.

To dissociate cytotoxicity and interaction, a common approach is to determine the sensitizer enhancement ratio, or reduction in dose needed to achieve a desired endpoint. To illustrate this for the experiments just shown, Figure 4 presents a compilation of data, where the left panel indicates the net response to CCNU of cells recovered from near the surface of spheroids, and the right panel, that of hypoxic cells deeper within the spheroid. The stars are the corresponding data points from those combination treatments shown in Figure 3, displaced downward from the predicted CCNU-only response by the 'excess' toxicity remaining after correction for the expected MISO toxicity. From this presentation, the combination treatment was

**Figure 3** Toxicity of misonidazole, CCNU, and the combination in spheroids and tumours. To achieve the comparable survival levels shown, spheroids in 5% $O_2$ were treated 30 min with 3.2 $\mu g\,ml^{-1}$ CCNU, and 2 h with 3.2 $mg\,ml^{-1}$ MISO; the tumour exposures were 4.0 $mg\,kg^{-1}$ CCNU and 1.0 $mg\,g^{-1}$ MISO. The products of the independent toxicities of the two agents are also shown.

**Figure 4** Toxicity of 30 min CCNU treatments to oxic, external (panel a) or hypoxic, internal (panel b) cells of spheroids, compiled from independent experiments (closed symbols are the data points also displayed in **Figure 3**). Additionally, the stars indicate the MISO + CCNU survival levels measured in the experiments plotted in **Figure 3**, but normalized to indicate survival relative to that expected for CCNU treatment alone (i.e., corrected for MISO toxicity). Isoeffective CCNU doses in the absence of sensitizer averaged 1.19 higher for cells from Fraction 2, and 1.44 higher for the internal cells.

about as toxic as a 20% greater CCNU dose in the aerobic fraction, and showed a further enhancement of only another factor of 2 in the hypoxic cells. Similar analysis of the tumour data results in less overall enhancement, and less differential between oxic and hypoxic cell populations.

A better estimate of the interaction would, of course, result from a more complete dose-response curve for the combination treatments. However, this would provide information at only one sensitizer concentration; we consequently prefer the 'median effect' analysis proposed by Chou and Talalay (1984) as a more general estimate of agent interaction that is also more easily extended to different exposure conditions.

The median effect equation allows a convenient mathematical representation of survival data, with broad applicability to most types of survival curves (Berenbaum, 1981; Chou & Talalay, 1984). The adequacy of the model can be assessed by evaluating the goodness of fit of 'transformed' data as in Figure 5 for spheroids; this plot essentially shows toxicity as a function of dose (each plotted point represents the mean of 1–3 independent experiments; unlike the previous figure, data are included for experiments where 'paired' determinations of combination to single agent toxicity were performed). Note that the representative data (fraction 2, near the outer rim of the spheroids, and fraction 8 near the necrotic region) were nicely fit by the model, and

**Figure 5** Spheroid survival data from MISO (squares), CCNU (circles), or combination treatments (triangles), transformed to allow solution of the Median Effect equation. Only data for two fractions (F2, open symbols, and F8, closed symbols) are shown; the best-fit linear regression curves of each data set are also indicated. Note the parallelism between the MISO and CCNU data for each fraction, and the upward displacement of the combination data, indicating that the response was potentiated.

the increased efficacy of each agent for the innermost cells was reflected by the increased slopes of the fraction 8 curves. The parallelism of the single agent curves (open and closed squares, or circles) indicates the legitimacy of adding the agent doses; the increased toxicity (upward displacement) of the combined modality data (1000:1 MISO:CCNU by weight) indicates a potentiated response.

The experimental data for all fractions through the spheroids were equally amenable to this analysis. Thus, it follows that a relatively simple interpolation between these dose-response curves (if sort fraction rather than depth in the spheroid is used in order to linearize the interpolation increments) leads to analysis of MISO/CCNU interactions in terms of the combination index, as in Figure 6. Note that in this three-dimensional representation, the interaction (combination index) between the agents is plotted on a logarithmic scale as a function of both the level of effect and of the position in the spheroid. The upper plane indicates an independent response (no interaction); all combination index values less than 1 indicate a synergistic interaction. As before, sort fraction 1 indicates cells on the periphery of the spheroid and fraction 10 is the 10% of the cells furthest from the surface. From this representation one can immediately appreciate that 'effective' doses of the two agents can lead to potentiation, and further, that the differential in interaction between the aerobic and hypoxic (outer and inner) cells of the spheroid was highly dependent upon the net treatment toxicity (i.e., the survival level). Like Figure 4, this analysis suggests, however, that the internal, hypoxic cells were only minimally more susceptible to chemopotentiation for most exposure conditions.

To determine whether the observed interaction between the agents was directly related to MISO metabolism and binding, the radioactivity recovered in each cell fraction of spheroids after addition of tracer levels of $^{3}H$-labelled misonidazole was plotted similarly in Figure 7. Note that for all fractions in the spheroids, MISO binding (incorporated $^{3}H$) was essentially a linear function of exposure concentration. As was expected, the hypoxic regions of spheroids bound more drug than the better oxygenated regions; this is the basis of using misonidazole (Chapman *et al.*, 1981) and other nitroheterocycles (Olive & Durand, 1983) as probes for hypoxic cells. If chemosensitization were directly correlated to the binding of MISO, the spheroid chemosensitization profile should be essentially the inverse of Figure 7 (which Figure 6 clearly is not). Our preliminary observations on $^{3}H$-MISO binding in tumours (unpublished), based on tumour disaggregation and sorting, produced results quite similar to the profiles shown for spheroids in Figure 7. Thus, the enhanced toxicity of the combined CCNU and MISO in tumours did not appear to correlate with MISO binding in tumours to any greater degree than in spheroids.

**Figure 6** The 'Combination Index' for interaction of misonidazole and CCNU in V79 spheroids, plotted as a function of depth in the spheroid (Sort Fraction 1 represents the outermost 10% of the cells), and survival level produced by the combination treatment. For reference, the upper plane is drawn at the level of independent action (antagonistic interactions were truncated at this level); increasing potentiation is indicated by decreasing values of the combination index. Note the constancy of the interaction with respect to cell position, despite the known variation in oxygen tension through the spheroid.

**Figure 7** $^3$H-misonidazole binding as a function of exposure concentration and position in the spheroid. Note that binding was very linear with exposure for each fraction, but that maximal binding at higher MISO concentrations was not found in the innermost cells of the V79 spheroids.

### Discussion

The data reported here indicate that chemopotentiation of CCNU toxicity by misonidazole occurred in *all* subpopulations of spheroid cells, and greater than additive cytotoxicity was observed in all cells of the KHT tumours. Moreover, the observed potentiation occurred to a surprisingly similar extent throughout each system, despite the fact that some cells are equilibrated with a 5% oxygen atmosphere, whereas others are at least orders of magnitude lower in oxygen content, if not completely anoxic. Since ploidy, cell cycle position, cycling rate, nutritional status, and similar variables change substantially through these subpopulations of cells, with little concomitant change in the degree of sensitization seen, it seems safe to conclude that these factors only minimally affect the net response.

In view of the somewhat different orientation of the spheroid and tumour experiments, the similarity of the conclusions reached with each system is gratifying. In particular, it is unlikely that the Hoechst staining 18 h after drug treatment identifies and locates exactly the same cells that were treated; the tumour is a dynamic system, with active proliferation in the aerobic regions, and continuous cell loss and replacement at greater distances from the functional vasculature. Nonetheless, our sorting techniques seem of particular value for hypoxia-targeted agents, since cell turnover might result in some underestimate of toxicity toward hypoxic cells, but would be expected to markedly underestimate aerobic toxicity and/or interactions.

It is clear from the conventional data presentation (Figure 3) that the observed cell kill from the combined treatments was greater than that which was expected on the basis of independent action of the two drugs. To go beyond that general statement, and to determine whether more cytotoxicity necessarily indicates greater potentiation, requires a more sophisticated analysis. The median effect analysis and the derived combination index leads to a specific definition of 'additivity', and identifies conditions under which the agents are sub-additive (antagonistic) or supra-additive (potentiating). Further, since the combination index is essentially the dose ratio for iso-effective treatments, an indication of the degree of *interaction* (as distinct from the level of cytotoxicity) is immediately available from this type of analysis.

Unfortunately, however, the median effect analysis also has some problems. Close scrutiny of Figure 5, which is the basis for the derived combination index, suggests that while the median effect model fits quite well for the single agent treatments, both combined modality treatments (triangles) would be represented better by concave upward curves (on the log-log plot). Forcing a linear curve through concave upward data points underestimates the degree of interaction at both low and high dose levels, and is thus quite sensitive to the dose-range over which the data are obtained. At low dose levels, this can produce apparent antagonism or, at best, an overestimate of the degree of antagonism present between the two agents. Conversely, at high doses, the effectiveness of the agents and the degree of interaction is underestimated. Both problems do, however, result in a conservative estimate of potentiation.

Use of the cell sorter to select different subpopulations of cells from the multicell systems, coupled with scintillation counting of bound $^3$H-misonidazole in the sorted cells, leads to the unique capability of correlating toxicity and drug exposure. Comparison, however, of Figures 6 and 7 suggests that the degree of interaction between MISO and CCNU (as quantified by the combination index) does not appear to be directly related to the sensitizer binding (and by inference, to the cellular oxygenation status). An analogous lack of correlation was observed when a nitrofuran, AF-2, was used as the chemosensitizer in spheroids (Durand & Olive, 1986), despite the fact that a good correlation was observed between AF-2 uptake and toxicity (Olive & Durand, 1983; Durand & Olive, 1986). It is important to note, however, that maximal toxicity *was* observed in the most hypoxic cells (Figure 3).

While the results reported here do not identify a 'specific' mechanism of chemosensitization, they do argue against 'complementary toxicity' being an important factor; both drugs show qualitatively similar toxicity profiles throughout the two systems, preferentially killing the more hypoxic cell populations in both. Thus, increasing the dosage of both agents might lead to 'wasted' activity, since the same cells are preferentially killed by each, and the potential for interactions would consequently be expected to decrease.

Other potential mechanisms of interaction include enhancement of DNA damage, reduction of glutathione and other endogenous protectors as a result of sensitizer treatment, and inhibition of repair of CCNU-induced damage. Again, one might expect that these mechanisms should be fairly closely coupled to the intracellular MISO concentrations, whereas the chemosensitization actually observed was clearly not (comparing Figures 6 and 7). Our data in the tumour system, showing enhanced CCNU toxicity in MISO-treated animals is, however, consistent with the expected result from MISO-induced elongation of the CCNU exposure time in animals (Workman *et al.*, 1983; Lee & Workman, 1986); the fact that the spheroid data so closely parallel the *in vivo* results argues that pharmacokinetic alteration seems unlikely to be the only mechanism operative *in vivo*. Thus, chemopotentiation is complex, and most likely, multifactorial.

In conclusion, we believe that our data indicate the need to perform chemosensitization experiments under highly quantitative conditions. Further, they suggest that the chemosensitization process is controlled or influenced by multiple factors, which will have to be carefully balanced in order to achieve optimum effects. Although the interaction between MISO and CCNU is clearly not dependent upon complementary toxicity, it would nonetheless seem advantageous if a chemotherapeutic agent which showed preferential toxicity to aerobic cells could in fact be chemosensitized; our preliminary data using misonidazole and melphalan (unpublished) suggest that both conditions can be achieved. As chemopotentiation may have considerable clinical potential, further studies addressing both mechanistic questions and improved drug combinations seem warranted.

This research was supported by the National Cancer Institute of Canada, and by NIH Grants CA-37879, and CA-40459. Technical assistance by Denise McDougal, Doug Aoki and Nancy Arnold is gratefully acknowledged.

### References

BERENBAUM, M.C. (1981). Criteria for analyzing interactions between biologically active agents. *Adv. Cancer Res.*, **35**, 269.

BROWN, J.M. (1982). The mechanisms of cytotoxicity and chemosensitization by misonidazole and other nitroimidazoles. *Int. J. Radiat. Oncol. Biol. Phys.*, **8**, 675.

CHAPLIN, D.J., DURAND, R.E. & OLIVE, P.L. (1985). Cell selection from murine tumour using the fluorescent probe Hoechst 33342. *Br. J. Cancer*, **51**, 569.

CHAPLIN, D.J., DURAND, R.E. & OLIVE, P.L. (1986*a*). Acute hypoxia in tumors: Implications for modifiers of radiation effects. *Int. J. Radiat. Oncol. Biol. Phys.*, **12**, 1279.

CHAPLIN, D.J., DURAND, R.E., STRATFORD, I.J. & JENKINS, T.C. (1986*b*). The radiosensitizing and toxic effects of RSU-1069 on hypoxic cells in a murine tumor. *Int. J. Radiat. Oncol. Biol. Phys.*, **12**, 1091.

CHAPMAN, J.D., FRANKO, A.J. & SHARPLIN, J. (1981). A marker for hypoxic cells in tumors with potential clinical applicability. *Br. J. Cancer*, **43**, 546.

CHOU, T.C. & TALALAY, P. (1984). Quantitative analysis of dose-effect relationships: The combined effects of multiple drugs or enzyme inhibitors. *Adv. Enz. Regul.*, **22**, 27.

CLEMENT, J.J., GORMAN, M.S., WODINSKY, I., CATANE, R. & JOHNSON, R.K. (1980). Enhancement of anti-tumor activity of alkylating agents by the radiation sensitizer misonidazole. *Cancer Res.*, **40**, 4165.

COURTENAY, V.D. (1976). A soft agar colony assay for Lewis lung and $B_{16}$ melanoma taken directly from the mouse. *Br. J. Cancer*, **34**, 39.

DEEN, D.F., HOSHINO, T., WILLIAMS, M.E. & 3 others (1980). Development of a rat brain tumor cell multicellular spheroid system and its response to 1,3-bis(2-chloroethyl)-1-nitrosourea and radiation. *J. Natl Cancer Inst.*, **64**, 1373.

DURAND, R.E. (1982). Use of Hoechst-33342 for cell selection from multicell systems. *J. Histochem. Cytochem.*, **30**, 117.

DURAND, R.E. (1983). Oxygen enhancement ratio in V79 spheroids. *Radiat. Res.*, **96**, 322.

DURAND, R.E. (1986). Use of a cell sorter for assays of cell clonogenicity. *Cancer Res.*, **46**, 2775.

DURAND, R.E. & OLIVE, P.L. (1986). Potentiation of CCNU toxicity by AF-2 in V79 spheroids: Implications for mechanisms of chemosensitization. *Int. J. Radiat. Oncol. Biol. Phys.*, **12**, 1375.

HALL, E.J. & ROIZIN-TOWLE, L. (1975). Hypoxic sensitizers: Radiobiological studies at the cellular level. *Radiology*, **117**, 453.

KELLY, J.P., HANNAM, T.W. & BILES, G.R. (1979). The cytocidal action of metronidazole in combination with other antineoplastic agents. *Cancer Treat. Rev.*, **6**, 53.

KWOK, T.T. & TWENTYMAN, P.R. (1985). The relationship between tumour geometry and the response of tumour cells to cytotoxic drugs – an *in vitro* study using EMT6 multicellular spheroids. *Int. J. Cancer*, **35**, 675.

LEE, F.Y.F. & WORKMAN, P (1986). Interaction of nitroimidazole sensitizers with drug metabolizing enzymes – Spectral and kinetic studies. *Int. J. Radiat. Oncol. Biol. Phys.*, **12**, 1383.

MOORE, B.A., PALCIC, B. & SKARSGARD, L.D. (1976). Radiosensitizing and toxic effects of the 2-nitroimidazole Ro-07-0582 in hypoxic mammalian cells. *Radiat. Res.*, **67**, 459.

MOULDER, J.E. & ROCKWELL, S. (1984). Hypoxic fractions of solid tumors: Experimental techniques, methods of analysis and a survey of existing data. *Int. J. Radiat. Oncol. Biol. Phys.*, **10**, 695.

MULCAHY, R.T. (1984). Effect of oxygen on misonidazole chemosensitization and cytotoxicity *in vitro*. *Cancer Res.*, **44**, 4409.

MULCAHY, R.T. (1986). Cross-link formation and chemopotentiatiation of EMT-6/Ro cells exposed to miso after CCNU treatment *in vitro*. *Int. J. Radiat. Oncol. Biol. Phys.*, **12**, 1389.

OLIVE, P.L., CHAPLIN, D.J. & DURAND, R.E. (1985). Pharmacokinetics, binding and distribution of Hoechst 33342 in spheroids and murine tumours. *Br. J. Cancer*, **52**, 739.

OLIVE, P.L. & DURAND, R.E. (1983). Fluorescent nitroheterocycles for identifying hypoxic cells. *Cancer Res.*, **43**, 3276.

OLIVE, P.L. & McCALLA, D.R. (1975). Damage to mammalian cell DNA by nitrofurans. *Cancer Res.*, **35**, 781.

ROSE, C.M., MILLAR, J.L., PEACOCK, J.H., PHELPS, T.A. & STEPHENS, T.C. (1980). Differential enhancement of melphalan cytotoxicity in tumor and normal tissue by misonidazole. In *Radiation Sensitizers: Their Use in the Clinical Management of Cancer*, Brady, L.W. (ed) p. 250. Masson: New York.

SIEMANN, D.W. (1984). Modification of chemotherapy by nitroimidazoles. *Int. J. Radiat. Oncol. Biol. Phys.*, **10**, 1585.

SUTHERLAND, R.M. & DURAND, R.E. (1976). Radiation response of multicell spheroids, an *in vitro* tumour model. *Curr. Top. Radiat. Res. Q.*, **11**, 87.

TAYLOR, Y.C., BUMP, E.A. & BROWN, J.M. (1982). Studies on the mechanism of chemosensitization by misonidazole *in vitro*. *Int. J. Radiat. Oncol. Biol. Phys.*, **8**, 705.

URTASUN, R.C., TANASICHUK, H., FULTON, D. & 4 others (1982). Pharmacokinetic interaction of BCNU and misonidazole in humans. *Int. J. Radiat. Oncol. Biol. Phys.*, **8**, 381.

WHEELER, K.T., WALLEN, C.A., WOLF, K.L. & SIEMANN, D.W. (1984). Hypoxic cells and *in situ* chemopotentiation of the nitrosoureas by misonidazole. *Br. J. Cancer*, **49**, 787.

WORKMAN, P., TWENTYMAN, P.R., LEE, F.Y.F. & WALTON, M. (1983). Drug metabolism and chemosensitization: Nitroimidazoles as inhibitors of drug metabolism. *Biochem. Pharmacol.*, **32**, 857.